# Motor Sequence Learning in Healthy Older Adults Is Not Necessarily Facilitated by Transcranial Direct Current Stimulation (tDCS)

**DOI:** 10.3390/geriatrics1040032

**Published:** 2016-12-05

**Authors:** Rachael K. Raw, Richard J. Allen, Mark Mon-Williams, Richard M. Wilkie

**Affiliations:** School of Psychology, University of Leeds, Leeds LS2 9JT, UK; r.allen@leeds.ac.uk (R.J.A.); m.mon-williams@leeds.ac.uk (M.M.-W.); r.m.wilkie@leeds.ac.uk (R.M.W.)

**Keywords:** Transcranial Direct Current Stimulation (tDCS), motor sequence learning, motor control, ageing, kinematic analysis

## Abstract

Background: Transcranial Direct Current Stimulation (tDCS) of the primary motor cortex (M1) can modulate neuronal activity, and improve performance of basic motor tasks. The possibility that tDCS could assist in rehabilitation (e.g., for paresis post-stroke) offers hope but the evidence base is incomplete, with some behavioural studies reporting no effect of tDCS on complex motor learning. Older adults who show age-related decline in movement and learning (skills which tDCS could potentially facilitate), are also under-represented within tDCS literature. To address these issues, we examined whether tDCS would improve motor sequence learning in healthy young and older adults. Methods: In Experiment One, young participants learned 32 aiming movements using their preferred (right) hand whilst receiving: (i) 30 min Anodal Stimulation of left M1; (ii) 30 min Cathodal Stimulation of right M1; or (iii) 30 min Sham. Experiment Two used a similar task, but with older adults receiving Anodal Stimulation or Sham. Results: Whilst motor learning occurred in all participants, tDCS did not improve the rate or accuracy of motor learning for either age group. Conclusion: Our results suggest that the effects of tDCS may be limited to motor performance with no clear beneficial effects for motor learning.

## 1. Introduction

Old age yields significant motor decline, including detrimental changes at a physiological level (e.g., loss in sensory sensitivity, weakening of muscles, and reduced flexibility of the joints [1,2,3,4,5]) and increased susceptibility to diseases that directly affect the motor system, such as stroke [6,7]. Age-related motor decline has profound outcomes, directly impacting an individual’s ability to complete activities of daily living (e.g., bathing, dressing, feeding) [8,9,10] and, in cases where disease disrupts and/or damages the motor system, movement can be lost entirely. This raises the question of whether there are therapeutic approaches that can help older adults retain or improve their motor skills. One potential approach is the use of electrical brain stimulation, with one study suggesting that anodal brain stimulation could help older adults compensate for decrements in motor skill [11].

The concept of applying electrical currents to the body for therapeutic benefit was initially supported by animal studies, which found that Direct Currents (DCs) could alter the response of neurons [12,13,14,15]. Since then, regions of the human brain have been stimulated, typically by applying saline-soaked surface electrodes on the scalp, and passing low amplitude DCs though the skull. In Transcranial Direct Current Stimulation (tDCS), the positive (anode) or negative (cathode) electrode is positioned over the area of interest (e.g., the primary motor cortex; M1) and another electrode is put over a reference region to complete the circuit. Once the electrical current penetrates the brain, it is thought that it can alter cortical excitability by modifying neuronal potentials and firing rates in response to stimuli [16,17]. Anodal DCs (atDCS) and Cathodal DCs (ctDCS) also produce opposing ‘polarity-specific effects’, where atDCS increases, and ctDCS decreases cortical activity (presumably by increasing or decreasing the likelihood of neuronal firing, respectively) [16,17,18,19,20,21,22,23,24,25,26,27].

Whilst there is evidence for the neurophysiological changes induced by tDCS directly beneath the stimulating electrode, this does not preclude wider ranging stimulation effects. Indeed, some imaging research suggests that the modulating outcomes of tDCS are not focused on one isolated region, but instead there is extensive impact across the brain [24,27]. The tDCS effects have yet to be clearly delineated [28], but some studies have endeavored to map boundaries [24,27]. In one such study, Lang et al. [29] observed altered bloodflow to brain regions well beyond M1 (when using atDCS and ctDCS over left M1), including changes in the right frontal pole, right primary sensorimotor cortex, as well as posterior brain regions. These broad effects imply that tDCS can influence cortico-cortical connections with an effect that spreads well beyond the brain regions immediately under the electrode [23,27,29,30].

The widespread stimulation effects should be perfectly suited to the requirements of rehabilitation therapies. For example, tDCS could be used with a patient who has experienced a classic left-hemisphere stroke to produce a general increase in cortical activity in the affected hemisphere (the hemisphere which controls the impaired contralateral limb that is to be moved during rehabilitation). Alternatively, ctDCS could be used in a similar way to Constraint Induced Movement Therapy (CIMT; i.e., an approach that promotes activation of the damaged cortex by encouraging patients to use the weaker limb in everyday activities, while constraining the stronger limb) [31], and applied to the non-affected hemisphere to inhibit use of the neural architecture that controls the unaffected limb [32,33]. Furthermore, because stroke patients often present with a number of different cognitive and motor problems, the broadly distributed effects of tDCS on M1 could serve to alter the excitability across many of the affected networks. This is especially important when a patient is trying to re-learn movements, because ‘learning’ (i.e., a change in internal processes through repeated practice of a motor behaviour, manifested in improved performance of that skill over time [34]), requires both a degree of motor function (e.g., the ability to move the limb in order to carry out an action) and the cognitive processes associated with learning the movement patterns (e.g., working memory and attention). Learning a new action will therefore not be achieved unless both the motor and cognitive systems are able to work together effectively. For example, our previous work has found that reduced motor performance can actually impact negatively on the processes necessary for learning a new sequence of movements, over and above the limits imposed by an individual’s cognitive capacity [35]. It appears that motor constraints (e.g., requiring use of the non-preferred hand to carry out the task) can impair complex motor sequence learning. If tDCS is capable of modifying brain activity within M1 (and beyond) then it has the potential to facilitate the learning of new movements.

Even though there is promise for the use of tDCS in the context of movement rehabilitation [36,37,38,39,40], the evidence-base is not sufficient to allow medical regulation authorities to recommend this approach within normal clinical practice. One review highlighted that despite reports that tDCS can boost general motor performance in patients with motor deficit following stroke, this does not translate to improvements in activities of daily living [10,41]. There is certainly some evidence that atDCS can improve contralateral motor performance (i.e., superior performance in the right hand when atDCS is applied to the left M1) in both healthy young [42,43,44,45,46,47,48] and older adults [11,49,50,51]. However, as has already been outlined, the essential process that needs to be enhanced during rehabilitation is motor learning, rather than motor performance. This will include the motor processes that initiate the movement itself, alongside a combination of higher-order cognitive elements such as reasoning and memory, which allow the procedural steps to be retained and retrieved [52,53].

Studies that have paired tDCS with motor learning tasks are limited in number. Some experiments have found facilitating effects of tDCS on motor learning (where performance improved over time) [54,55,56], whereas other attempts have failed to show any benefit at all [57,58,59]. When the process of learning a new motor skill has been split into its constituent stages—namely ‘learning’ (a gradual increase in performance across trials) and ‘consolidation’ (when performance stabilises and the learned behaviour is consolidated to memory), some studies have suggested that atDCS is capable of facilitating both components, firstly by strengthening newly formed associations in the brain (i.e., during learning); and secondly by improving the formation of a memory (i.e., during consolidation) [25,60]. A recent study was unable to support these suggestions, however, reporting no impact of atDCS on the retention of new motor memories in healthy participants [61]. Cathodal Stimulation (CS) of the cerebellum did, nevertheless, inhibit the formation and retention of a new motor memory.

Not only is the tDCS literature plagued with an unresolved mixture of results (and a strong likelihood of a ‘bottom-drawer effect’ with regards to null findings), a further issue with the evidence-base is that the motor performance measures taken in tDCS studies are often limited; it is common for single metrics of speed or accuracy to be recorded during basic circle drawing, handwriting, grip force or finger-sequencing tests [41,42,43,44,45,46,47,48,49]. This approach means that either no insight is gained into the effects of tDCS on the ‘quality’ of motor performance (i.e., in studies where participants are merely timed), or it is only demonstrated how accuracy is affected, in the absence of information on interactions with movement speed (i.e., whether there was a speed–accuracy trade-off). The Jebsen–Taylor Hand Function Test (JTT; a measure of everyday hand functions including writing and simulated feeding) [62] is probably the most diverse of all the tasks used during or post tDCS, because it includes multiple tasks within both fine and gross motor subsets (i.e., fine = turning cards, grasping small objects, lifting small objects with a spoon; gross = stacking checkers and lifting light/heavy cans). It is still the case, however, that a single outcome measure is taken (time in this case, with no metric of accuracy included). This is particularly problematic when testing older adults, since they often make compensatory changes to the spatial/temporal dynamics of their movements to compensate for motor decline [63,64]. For this reason, it can be argued that a measure of spatial accuracy is essential when studying movement in older groups [35,65,66,67].

The problem with over-simplified tasks also arises in many of the studies showing beneficial effects of tDCS on ‘learning’, since the tasks often have limited motor complexity, and frequently adopt a modified version of a simple Serial Reaction Time Task (SRTT). In the traditional SRTT [68], participants use four buttons to respond to one of four lights that appear in a repeated or random sequence. A quicker reaction time in the repeated condition is used as a measure of ‘implicit’ (unconscious) learning, whereas a faster reaction time for the random sequence reflects general improvements in motor response, irrespective of learning (where planning of movements based on prior experience is minimised). One can argue that this type of task is not particularly engaging, because participants are not making a conscious effort to learn a new skill. Moreover, increased reaction times are used as the metric of motor learning, which is not necessarily the main variable of concern during movement rehabilitation (i.e., where spatial error is often the priority). Indeed, when tDCS has been combined with visuomotor tracking and grip force learning tasks, only limited beneficial behavioural effects on motor performance or learning have been observed [58,59,69]. In Saiote et al.’s study [58], participants had to grasp a ball and adjust the level of pressure applied, to control the visual stimuli that moved on a display screen. Learning was measured by computing tracking error (i.e., the difference between the correct pressure and the pressure applied by the participant) and analysing how this changed as the task progressed. The lack of tDCS effects suggests that atDCS may not enhance the learning of tasks that require sustained attention to improve spatial accuracy.

It should be acknowledged that quantifying task engagement is non-trivial but it appears that the type of set-up used by Saiote et al. [58] demands a greater degree of attention resources relative to measures of repetitive finger-tapping movements. The fact that tDCS failed to facilitate learning in Saiote et al.’s [58] study, despite previous positive reports [54,55,56], also implies that the effects may depend on the complexity of the experimental task. It appears, therefore, that the evidence to support the use of tDCS in a rehabilitation setting is insufficient. Even though some research with young adults suggests tDCS may enhance learning of basic motor skills [54,55,56], this finding has not been widely replicated with more complex learning tasks [58,59,69]. More concerning still, surprisingly few studies have tested tDCS with healthy older adults who could benefit from an intervention that boosts motor performance and/or learning (i.e., due to their reduced motor skills [11,35,65,66,67]).

In order to address the underrepresentation of older adults in the tDCS literature, and improve the over-simplified motor tasks used in previous experiments [11,41,42,43,44,45,46,47,48,49,50,51,54,55,56], the present work applied a novel sequence learning design that was sensitive to cognitive and motor differences across younger and older adults [48]. This task allowed an investigation of whether tDCS enhances motor performance and/or sequence learning across different age groups by measuring the movement speed, movement accuracy as well as the rate of motor learning. In Experiment One, young right-handed adults learned a sequence of 32 aiming movements using their preferred hand, whilst undergoing one of three tDCS conditions; atDCS of the left (i.e., dominant) M1, ctDCS of the right (i.e., non-dominant) M1, or sham (S) stimulation. If tDCS is capable of accelerating complex motor sequence learning, both the active stimulation conditions (i.e., atDCS and ctDCS) should improve learning relative to sham; (i) Anodal Stimulation (atDCS) should work by increasing the excitability of the left hemisphere, thus having a positive impact on the motor behaviour of the right hand, whereas right-sided ctDCS should improve motor learning through reduced inhibition of the left hemisphere, essentially by dampening the effects of interhemispheric inhibition [16,18,27,41,42,43,44,45,46,47,48,49,50,51]. Alternatively, if tDCS is unable to modulate the network of cognitive and motor processes involved in our experimental task, similar rates of learning across all stimulation conditions would be expected. In Experiment Two, we used a similar learning task with older adults (who usually exhibit slower and less accurate movements than the young). It might be predicted that tDCS would be more effective with older participants, because age-related cognitive and motor provides a greater opportunity for learning to be enhanced [11].

## 2. Method and Materials

### 2.1. Experiment One

#### 2.1.1. Participants

Twenty-five healthy adults (15 female, 10 male) aged 21–35 years (mean age = 26.32, *SD* = 4.56) were recruited from an opportunistic sample. All participants were right-handed, as indicated by scores on the Edinburgh Handedness Inventory (Mean EHI = 91.72; *Standard Deviation*: *SD* = 15.18). To determine whether participants were fit and healthy to undergo Transcranial Direct Current Stimulation (tDCS), a Medical Health Questionnaire (MHQ) was administered, whereby individuals were not recruited if they (i) had a history of ophthalmological or neurological problems; (ii) had experienced faintness, light-headedness, blackouts, severe headaches, unusual heartbeats/palpitations in the last 12 months; (iii) had ever undergone electro-convulsive therapy; (iv) were pregnant; (v) had a personal or family history of epilepsy; (vi) had in the past experienced head trauma with loss of consciousness; (vii) had any metal fragments present in their body (this included previous injury with a metallic foreign body, or a prior engagement in metal grinding); (viii) had a medical device implanted in their head (including any type of bio stimulator, internal electrodes, electronic, hearing aids, eye prostheses, dentures, or any other electrical, mechanical or magnetic implant). Suitable candidates were randomly assigned to one of three conditions based on the nature of brain stimulation to be received; atDCS (*n* = 9), ctDCS (*n* = 10) or S (*n* = 6) tDCS. The procedure for randomisation involved assigning participants at the time of booking an appointment to take part, to atDCS (participant one), ctDCS (participant two) and S (participant 3), in this order, on a repeated basis. The reason that fewer participants were recruited into the atDCS and S groups was because these participants cancelled their appointments to attend, following assignment to a condition. The University of Leeds ethics and research committee approved this experiment (April 2011) and all participants gave written, informed consent in accordance with the Declaration of Helsinki (NB. this also applied to Experiment Two).

#### 2.1.2. Motor Sequence Learning Task

**Figure 1:**
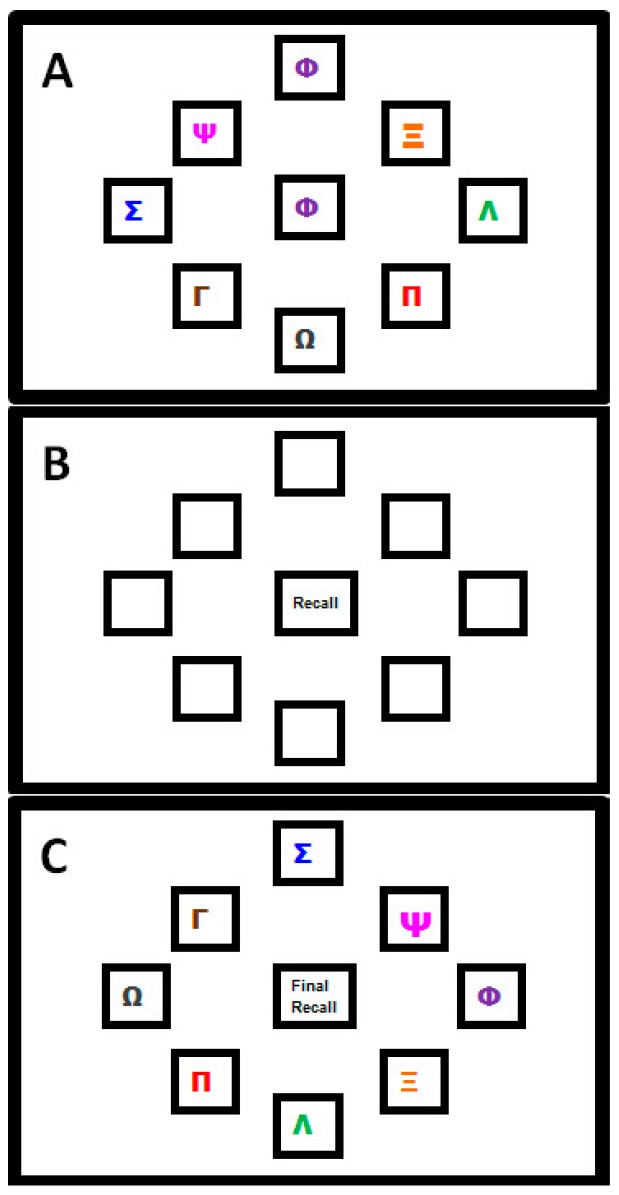
Motor Sequence Learning Task in Experiment One. (**A**) Training Trial: where participants moved the stylus into the box corresponding to the Greek letter that appeared in the centre; (**B**) Test Trial: in which participants attempted to recall the pattern of movements they had just practiced but without letters visible; (**C**) Transfer Trial: where Greek letters were rotated two positions clockwise from their position in the Training Trial and participants had to recall the sequence order by moving to new locations on the screen (NB. this trial followed the final Test Trial; this image is not to scale).

A complex motor sequence learning task was created using the kinematic software ‘KineLab’ [70]. The task was designed to occupy participants for roughly the full duration of the 30 min tDCS intervention period (though the exact task time varied between participants), and participants used a handheld stylus (stylus length = 150 mm; nib length = 1 mm) to interact with stimuli presented on a tablet Personal Computer (PC; screen width = 260 mm; screen height = 163 mm). The aim of the task was for participants to learn a sequence of aiming movements made with their preferred (right) hand to eight target locations on the screen. Fourteen ‘Training’ and ‘Test’ trials alternated, allowing participants to practice and reproduce the sequence repeatedly (i.e., Training trial, then Test trial × 14 repetitions = 28 trials in total). Figure 1 shows the Training trial, with a central white box (height = 25 mm; width = 25 mm) surrounded by eight ‘target’ boxes (height = 25 mm; width = 25 mm), each containing a different coloured letter of the Greek alphabet. In the Training trials, one of eight target letters appeared in the central box for 1 s as a cue for participants to move the stylus to the target box containing the same letter (e.g., move from the centre to the purple Phi in Figure 1a). After each individual move to a target box, participants returned to the centre (without clicking the mouse at any point), where the next letter in the sequence would appear. There were 32 letters in the sequence, which was the same for every Training trial (i.e., the aim was to improve recall of the same 32-move sequence). After each Training trial, a Test trial required participants to reproduce the sequence of moves they had just been practicing (i.e., move the stylus back-and-forth between the central box and target locations as quickly and as accurately as possible), but without the letters visible on the screen; see Figure 1b). Participants were given verbal instructions whereby they were asked to “complete the task as quickly and as accurately as possible”.

The task was designed specifically for the following reasons: (i) it demands a large number of moves to be retained through repetition, hence we believe participants were more likely to remain engaged (i.e., develop a motivation to learn a few more moves per Test trial, and ‘rise to the challenge’ of trying to remember such a long sequence); (ii) it requires motor accuracy, sustained attention and cognitive processes (e.g., working memory and associative learning—we assume this, given the fact that older adults show reduced performance on our test [35], making it more complex than the SRTT paradigms used in past studies [55,56]; (iii) it is arguably more reflective of learning in the real-world, where we are accustomed to interacting with objects that have a number of salient properties which can vary such as shape, colour, size and location (e.g., using the spatial location of the numbers on an Automated Teller Machine (ATM) keypad as cues to recall your password); (iv) it made it possible to test how participants were learning the sequence, i.e., whether participants would learn the sequence of colours and symbols, or the spatial location. Greek letters were chosen (rather than Roman characters) as a convenient set of diverse symbols that would not be trivial to articulate, and would not create word-like strings. Note that none of the participants in this study spoke or read Greek, nor did they have background knowledge to suggest regular use of the Greek alphabet in their daily lives—it is therefore unlikely that a strategy of translating shapes to letter was employed by our participants when trying to learn the sequence). To test whether participants were learning the spatial location of the target letters, or instead using some feature characteristic such as shape or colour of symbols, a ‘Transfer’ trial was included at the very end of the task. This trial prompted participants to recall the sequence when the symbols inside the target boxes had all been rotated two positions clockwise from their original placement in the Training trial set-up (i.e., participants had to move the cursor to the same target but in a new location, maintaining the sequence order that had been practised). If participants were learning the spatial locations of the letters, they would find it difficult to reproduce the sequence when the locations had changed. This trial challenged participants to exhibit a high degree of concentration in order to override the mental representations of target locations in order to move to the correct target, rather than to the location where the target had been appearing repeatedly throughout the main task.

To ensure that participants had a complete understanding of the task, standardised instructions were given in a short visual presentation on the PC, which included pictures of the three trial types (similar to Figure 1a–c), and participants had the opportunity to practice the different trial types which featured a 16-element sequence different to that used in the experimental task. One concern about this practice test is that it could have introduced ‘proactive interference’, whereby exposure to the short 16-element sequence might have hampered learning of the longer sequence [71,72]. Nevertheless, it was vital that participants had a firm understanding of the task for learning to occur, and it was not a task that was easy to explain without visual examples (e.g., the different trial types had various ‘rules’ associated with their completion, such as returning to the central box between moves, and omitting any mouse clicks). It is for this reason that we felt it worth the risk of any potential ‘interference’, to avoid participants’ confusion over how the task should be executed.

#### 2.1.3. Procedure for Transcranial Direct Current Stimulation (tDCS)

Transcranial Direct Current Stimulation (tDCS) was delivered from a battery-powered constant current stimulator (Magstim™ Eldith model), using a set of two rubber electrodes (25 mm^2^) covered with saline-soaked sponges (manufactured by NeuroConn, Ilmenau, Germany). The electrode pads were re-hydrated in a standardised manner between each new participant: the pads were left in shallow trays of saline solution for 30 min prior to use, whereby the depth of the solution was sufficient enough to cover the whole pad. Excess saline was removed before they were used so they did not drip. The quantity of saline solution absorbed by the electrode pads was 20 mL. The tDCS stimulator used in this study is widely used in labs around the world, has a maximum current of 5000 µA (±1%) and is able to deliver stimulation for up to 30 min. For the purpose of this study, a program was set to deliver 30 min of constant current stimulation at an intensity of 1.5 mA. This would be equal to a stimulation intensity of 2 mA had larger 35 cm^2^ electrodes been used). This included a ‘ramp-up’ and ‘ramp-down’ period of 30 s (i.e., the current took 30 s to gradually increase and a further 30 s to decrease at the start and end of the testing period respectively). A Sham (S) condition was also programmed to deliver 60 s of stimulation at 1.5 mA, in between a 30 s ramp-up and ramp-down period. While the current intensity could have been set higher at 2.0 mA, a pilot test with one participant found that 30 min of stimulation at this level was uncomfortable; in that it led to increased skin temperature, redness and mild blistering. This outcome was based on verbal reports from the single participant involved in the pilot session, and observations of skin colour/condition made by the researcher. When the participant was asked to describe the complaints with regards to the procedure, the participant said that the electrode felt ‘hot’ on the skin. Furthermore, the experimenter administering the tDCS reported seeing a dark pink 5 mm blister on scalp of the participant beneath the active anode, which was described by the participant as being ‘sore’ but not painful’.

A current of 1.5 mA was found to be tolerable and did not cause reported side effects. The International 10/20 system of electrode placement was used to locate the brain region of interest depending on the stimulation condition; atDCS of the left M1, ctDCS of the right M1, or S, whereby the positioning of electrodes for atDCS and ctDCS was counterbalanced across participants. The reference electrode was always placed above the contralateral supraorbital area (i.e., the part of the forehead above the eye on the opposite hemisphere to the stimulating electrode). We chose to stimulate M1 because we believe motor performance to be an essential pre-requisite of motor learning, thus if tDCS were to improve the quality of movement, it should in turn increase a person’s ability to learn a new set of movements. The electrodes were secured with two rubber straps that wrapped over and around the head to ensure optimal contact with the skin. To ensure that the electrodes remained tight to the scalp and sufficiently soaked throughout the experimental task, participants were not prepped for tDCS until after they had received the instructions for the motor sequence task and completed the practice trials. Participants were also given 30 s after the initial ramp-up in order to accommodate to the sensation of tDCS before beginning the task. For the purpose of the motor task, participants were seated at a table with the tablet PC placed at a comfortable distance in front of them. The experimenter who delivering the tDCS and computerised task was not blinded to the tDCS condition.

#### 2.1.4. Analysis

The aim of Experiment One was to establish whether tDCS could enhance learning of a complex motor sequence by young adults. The following outcome measures were calculated for each Test trial:

(i) *Sequence Learning Measure:* Number of moves recalled in the correct sequential order (i.e., Correctly Recalled; CR), with a maximum score of 32. Points were not deducted for incorrect moves;
(ii) *Movement Speed Measure:* Recall Movement Time (MT), the mean time (s) taken to move the mouse from the centre to a target box when recalling the sequence, was taken as an indication of motor performance.

Mean values across the First Five (F5) and Last Five (L5) Test Trials were calculated for the two outcome measures, with change in performance providing an indication of learning. Separate mixed-model ANOVAs for each metric (CR and MT) compared F5 and L5 performance for the three stimulation groups (i.e., atDCS, ctDCS and S). In the final Transfer condition, the spatial positions of the target letters were rotated, and the same metrics taken. Two ANOVAs were conducted on CR and MT to compare outcomes in the Transfer trial with the final Test trial, for the atDCS, ctDCS and S conditions. For each ANOVA, we report the F Statistic (*F*; the F ratio of explained variance to unexplained variance), P value (*p*; the calculated probability of finding a value equal to or more extreme than actually observed), and the partial eta squared (*η^2^_p_*). Across all ANOVAs, the Greenhouse-Geisser estimates of sphericity (ε) are reported where degrees of freedom have been adjusted.

#### 2.1.5. Results: Test Trials

**Figure 2:**
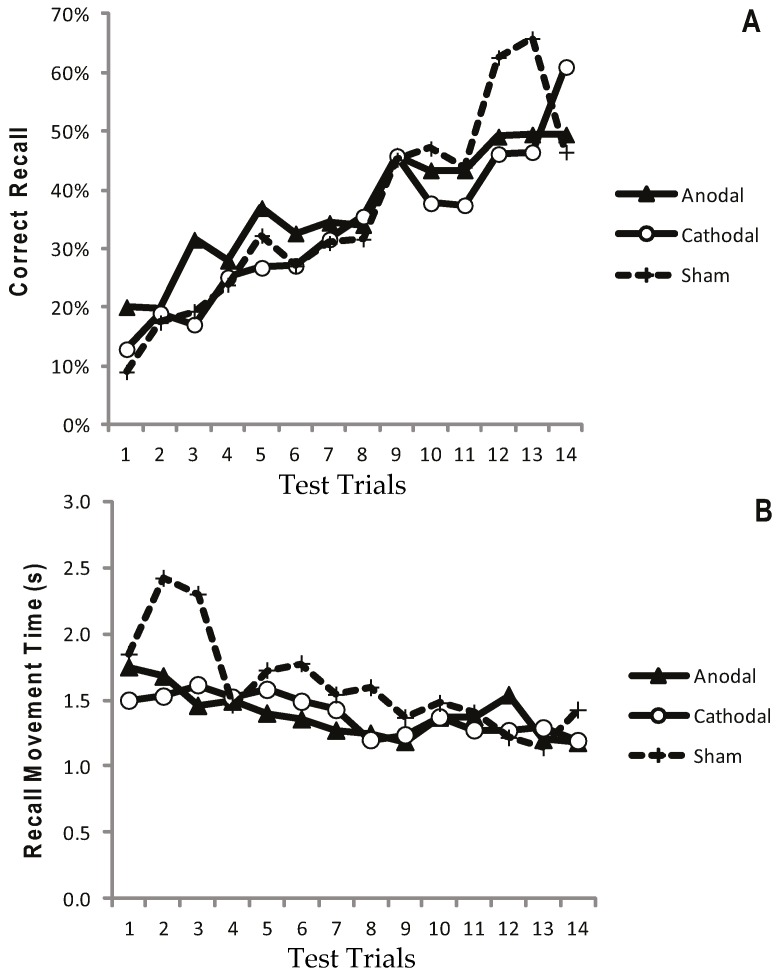
Progressive increase in Correct Recall and decrease in Recall Movement Time with no apparent differences between treatment arms. Measurements of Motor Learning in Experiment One (All Trials). Motor learning measures for the Anodal (atDCS; Filled triangle symbols), Cathodal (ctDCS; Open circle symbols) and Sham (S; dashed line, cross symbols) stimulation groups for each of the 14 Test Trials. The x axis shows trial number. (**A**) Proportion (%) moves recalled in the correct sequential order (CR) (**B**) Mean time taken between moves during free recall (MT).

**Figure 3:**
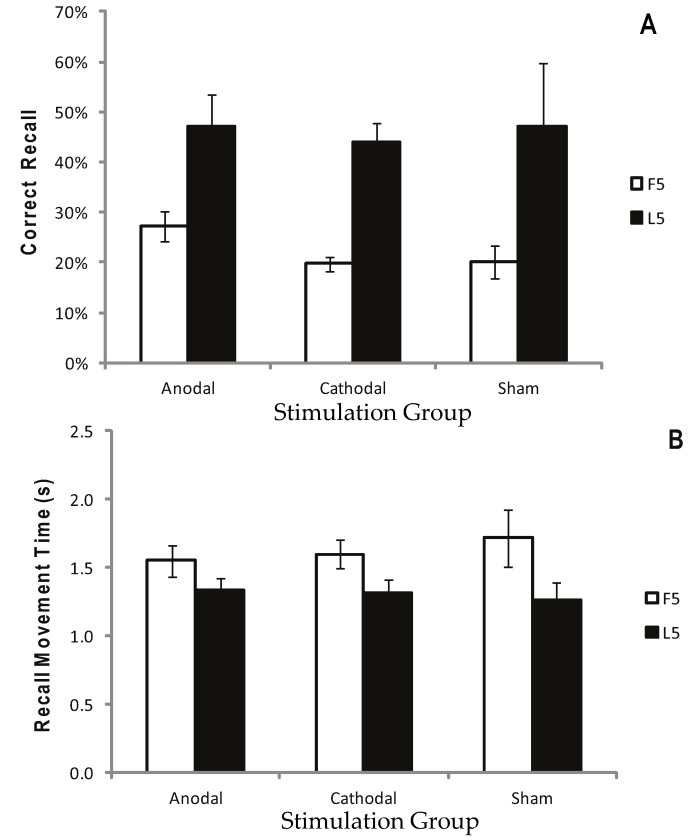
Correct recall and recall movement time showing no reliable difference between tDCS and sham. Measurements of Motor Learning in Experiment One (First Five vs. Last Five Trials)**.** Motor learning measures for the Anodal (atDCS), Cathodal (ctDCS) and Sham (S) stimulation groups averaged across the First Five (F5; white bars) and Last Five (L5; black bars) Test Trials. (**A**) Proportion (%) of moves recalled in the correct sequential order (CR) (**B**) Mean time taken between moves during free recall (MT). Bars = standard error of the mean.

Figure 2a displays the mean CR for the atDCS, ctDCS and S conditions. Participants remembered more of the sequence as the trials progressed, and there was a significant increase in CR between the F5 and L5 trials (*F* (1, 22) = 40.41, *p* < 0.001, *η^2^_p_* = 0.65; Figure 2a and Figure 3a). There was, however, no main effect of stimulation group (*p* = 0.748), and no trial × stimulation group interaction (*p* = 0.709). This suggests that tDCS had no facilitating effect on the number of moves participants were able to recall.

Recall Movement Time (MT) data revealed a gradual increase in the speed at which participants made their movements across the duration of the task (see Figure 2b and Figure 3b). A main effect of trial block also showed a significant decrease in MT between the F5 and L5 trials (*F* (1, 22) = 23.9, *p* < 0.001, *η^2^_p_* = 0.52), suggesting that participants were able to recall the moves faster with practice (Figure 2b and Figure 3b). There were no effects of stimulation group (*p* = 0.882), and no trial × stimulation group interaction (*p* = 0.376).

#### 2.1.6. Results: Recall in the Transfer Trial

**Figure 4:**
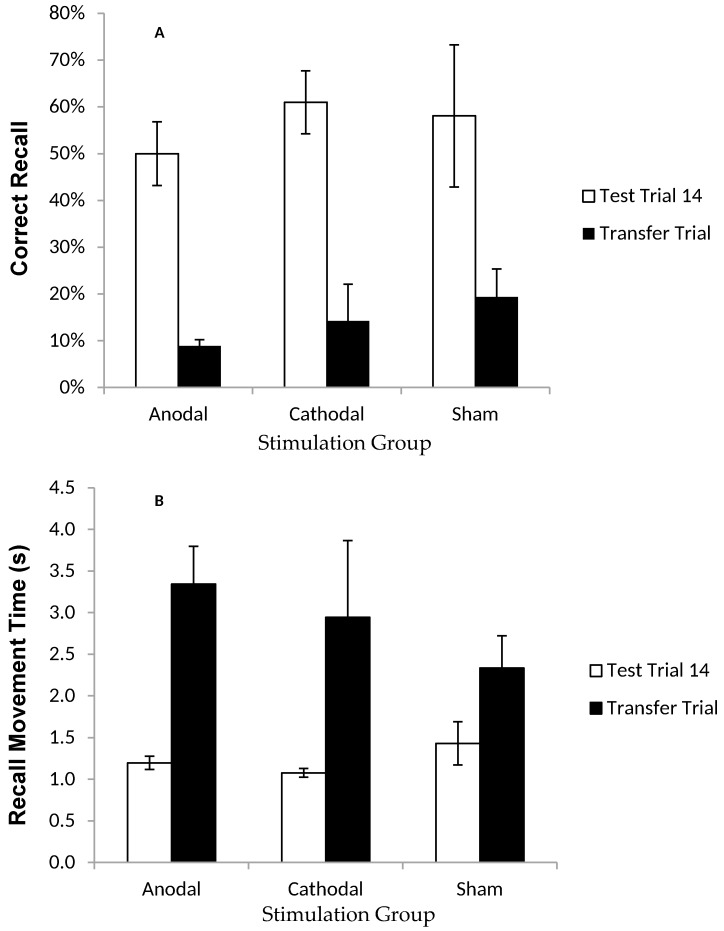
Correct recall and recall movement time showing no reliable difference between tDCS and sham groups in the transfer trial. Measurements of Motor Learning in Experiment One (Last Test Trial vs. Transfer Trial). Motor learning measures in the final (14th) Test Trial (white bars) and the Transfer Trial (the trial following the final Test Trial; black bars) for Anodal (atDCS), Cathodal (ctDCS) and Sham (S) stimulation groups. (**A**) Proportion (%) of moves recalled in the correct sequential order (CR) (**B**) Mean time taken between moves during free recall (MT). Bars = standard error of the mean. Bars = standard error of the mean.

In the Transfer trial, participants had to recall the sequence on a screen where the target letters were visible, but rotated two positions clockwise from their original location in the Training trials (Figure 1c). By comparing CR and MT between this Transfer trial and the last Test trial, it was possible to establish whether participants were simply learning the order of letters/colour, or their spatial locations. ANOVAs comparing the last test trial and the transfer trial showed a significant decline in movement speed (i.e., a main effect of trial on MT; (*F* (1, 16) = 17.72, *p* < 0.05, *η^2^_p_* = 0.53) and accuracy (i.e., a main effect of trial on CR; *F* (1, 21) = 46.89, *p* < 0.001, *η^2^_p_* = 0.70) of recall (Figure 4a,b). There was no effect of stimulation group on CR (*p* = 0.506) or MT (*p* = 0.520), or a trial × stimulation group interaction on CR (*p* = 0.225) or MT (*p* = 0.476).

#### 2.1.7. Discussion

The first experiment examined whether tDCS would accelerate learning of a complex motor sequence. The first issue was to examine the nature of the learning experienced by participants. Patterns of performance were typical of a novel task—learning curves were exhibited whereby participants remembered more of the motor sequence as the task progressed. It was a challenging task with only around half the 32 moves retained by the final Test Trial; though participants did become quicker at recalling the moves in the second half of the task (i.e., as indicated by lower MT values); poor performance in the Transfer condition (CR scores dropped dramatically, and MT increased) suggests that participants were learning the spatial locations of the Greek letters rather than just the order in which the colours/letters appeared.

Whilst the results confirm that participants were learning to move to a series of targets in a spatial sequence, the critical issue was whether tDCS would enhance some aspect of learning: in particular, ctDCS (of the right M1) should, according to some previous studies, cause superior right-hand performance relative to S [54,55,56]. However, no significant differences were found between the three simulation groups on either of the measures of motor learning in the current experiment; there was no effect on the number of moves recalled in the correct order, and no effect on speed of recall. Thus, even though participants showed progressive learning of the task, there was no evidence to suggest that tDCS had any beneficial effect on this improvement. In addition, tDCS had no impact on the outcome of the Transfer trial, with all participants appearing to have encoded the sequence spatially, regardless of stimulus group (hence no stimulation group × trial interaction found for CR or MT when comparing the Transfer trial with the final Test trial).

A possible explanation for the absence of tDCS effects in Experiment One could relate to the age and skill level of the participant group. Participants in the experiment were young, well-educated, university students (mean age = 26 years), who were likely to perform at a high-level and already had an excellent capacity for engaging and learning new skills. It might therefore be that there was little room for improving learning within this population [11,42]. For instance, Boggio et al., [42] found that participants’ performance on the JTT was improved by atDCS of the right M1 when the non-preferred hand was used, but not when tDCS was applied to the left M1 when the preferred hand was used. The authors attributed this to the fact that ‘under-use’ of the non-preferred hand in daily life means that the non-preferred M1 can benefit from tDCS. Stimulation of the left (i.e., dominant) cortex, however, leads to a ceiling effect and no behavioural improvement [11], as this hemisphere is already optimally activated. To establish whether tDCS is more likely to improve motor learning in a population where the brain would benefit from cortical stimulation, we ran a second experiment with older adults (who typically show an age-related decline in motor performance and learning [11,35,65,66,67,73]). In a recent experiment, tDCS was found to boost motor learning sufficiently to allow older adults to compensate for their natural motor decline; though the same effect was absent in a younger (and arguably more capable) group, who did not benefit from the intervention [11].

### 2.2. Experiment Two

Experiment One found no effect of tDCS on the number of moves young adults were able to learn of a novel motor sequence, nor on movement times. Experiment Two used a similar learning task to examine whether tDCS would improve learning in an older population, who might be predicted to show worse motor learning [35,73]. As no differences were found between the atDCS and ctDCS groups in Experiment One, Experiment Two only compared the effects of atDCS of left M1 with S stimulation. Note that we are aware that as a consequence of healthy ageing and plasticity, the older brain undergoes structural and functional changes [74], which could have implications with regards to the effect of CS over the non-dominant right hemisphere in older individuals. The decision was made to omit one of the active stimulation conditions because of the difficulties in recruiting healthy older adults for experimental research—especially older adults clear of neurological medical history and/or ophthalmological problems. Given the paucity of studies that have used the technique in older groups, we also did not want to potentially put at risk an arguably more vulnerable group of individuals (some of our participants were over 80-years-old). Furthermore, when deciding which tDCS condition to repeat in the second experiment, we chose to keep atDCS in the study because this is the condition most commonly used within the literature, and we were particularly interested in examining a technique that could increase the potential of neuronal firing in order to improve motor function (i.e., as opposed to dampening inter-hemispheric inhibition as claimed by those who suggest the efficacy of ctDCS).

#### 2.2.1. Participants

Seventeen healthy adults (eight females, nine males) aged 60–85 years (mean age = 69.82, *SD* = 8.47) were recruited from a new opportunistic sample. All participants were right-handed (mean EHI score = 96.31, *SD* = 8.48). The MHQ (see Experiment One) was used to determine whether participants were healthy with no conflicting medical conditions that would exclude them from being able to receive tDCS (as outlined in Experiment One). Suitable candidates were assigned to one of two brain stimulation conditions, whereby participants one-to-ten received active atDCS and participants eleven-to-seventeen were allocated to the S condition.

#### 2.2.2. Motor Sequence Learning Task

**Figure 5:**
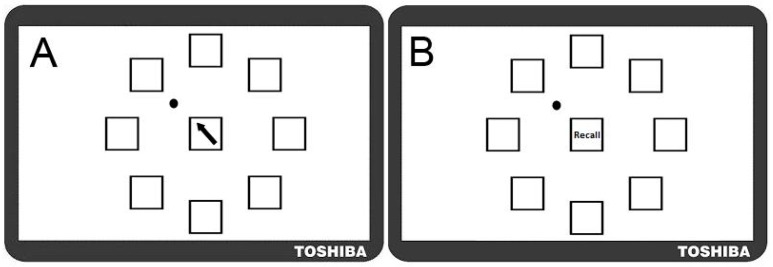
Motor Sequence Learning Task in Experiment Two. Screen shots of the modified motor sequence learning task as it appeared to participants (not to scale). (**A**) Training Trial in which participants moved the dot (top left pictured) according to directional cues that appeared in the central box; (**B**) Test Trial in which participants recalled the pattern of movements they had previously practiced displayed in the training trial.

Experiment Two involved a modified version of the motor sequence learning task used in Experiment One. Participants used the same tablet PC and a standard computer mouse (with their preferred right hand) to learn a series of movements made to eight possible target locations on the screen. One might argue that the use of a computer mouse could be slightly less intuitive for the older adult population. Our own observations of older participants interacting with the tablet laptop during pilot testing, suggested that this population was comfortable with this level of technology, and accordingly, none of the participants in the present study had any difficulty engaging with the equipment. Fourteen Training and Test trials alternated to allow participants to practice and then reproduce the sequence repeatedly (i.e., Learning trial followed by a Test trial repeated 14 times = 28 trials in total). In each Training trial, there was one central white box (height = 25 mm; width = 25 mm), surrounded by eight identical target boxes (Figure 5a), and a black arrow appeared in the central box as a cue for participants to move the cursor to the target location adjacent to the direction of the arrowhead (e.g., top left in Figure 5a). After each individual move to a target location, participants returned the cursor to the centre, where the next arrow in the sequence would appear. There were a total of 30 moves to learn, which followed a random pattern (i.e., the same pattern for every Training trial). In the following Test trial, participants had to reproduce the sequence of moves they had just been practicing, by moving the cursor back-and-forth between the central box and target locations as “quickly and as accurately as possible” (Figure 5b). To ensure that participants understood the task, we administered a short presentation with pictures of the two trial types (similar to Figure 5a,b). Participants were also given one practice each of a Training and Test trial featuring a 16-move sequence different to that used in the experimental task. The task lasted between 35–40 min and therefore typically occupied participants just beyond the full 30 min tDCS intervention period.

Note that the learning task was modified from Experiment One (which used Greek letters), to ensure that the visual stimuli were clearly visible to older adults, who often have some degree of visual impairment (e.g., difficulty distinguishing between colours symbols). The use of clear black arrows provided reassurance that participants’ performance did not reflect difficulty in recognising the stimuli. Given that Experiment One established that the spatial layout was the predominant characteristic learned, (despite the presence of other cues), it was expected that the new task would tap into similar cognitive and motor capabilities as those used previously.

#### 2.2.3. Procedure for Transcranial Direct Current Stimulation (tDCS)

The same protocol for the delivery of tDCS, and an identical set-up for completion of the learning task, was used as outlined in Experiment One. After the task was explained and practised, participants underwent either atDCS or S as they completed the task. Cathodal Simulation (ctDCS) was not used in Experiment Two and the experimenter was not blinded to the experimental condition.

#### 2.2.4. Analysis

Outcome measures were identical to those in Experiment One. Mean values for CR (out of a maximum of 30 moves) and MT across the F5 and L5 Test trials were calculated and separate mixed-model ANOVAs were conducted to examine differences in motor learning and speed of recall between the A and S conditions.

#### 2.2.5. Results

**Figure 6:**
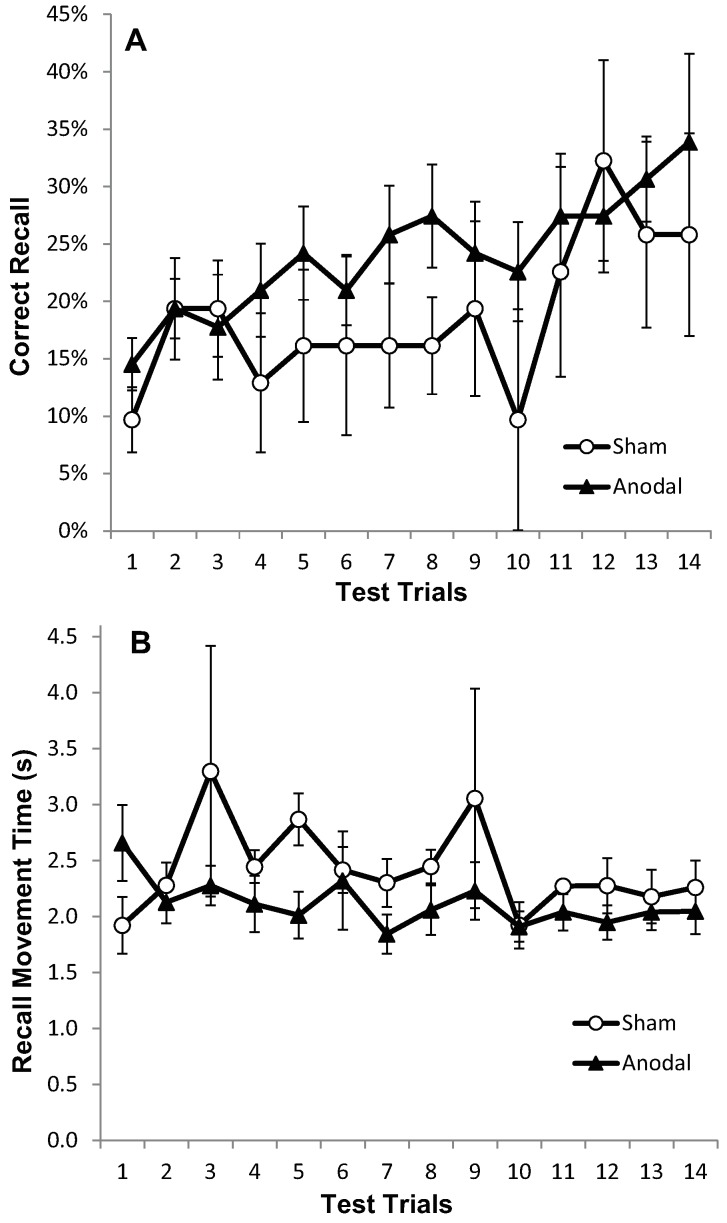
Correct recall and recall movement time showing no reliable differences between tDCS and sham in older adults. Measurements of Motor Learning in Experiment Two (All Trials). Motor learning measures for the Anodal (atDCS; black triangles) and Sham (S; white circles) stimulation groups for each of the 14 Test Trials (**A**) Proportion (%) of moves recalled in the correct sequential order (CR) with standard error bars (**B**) Mean time taken between moves during free recall (MT).

**Figure 7:**
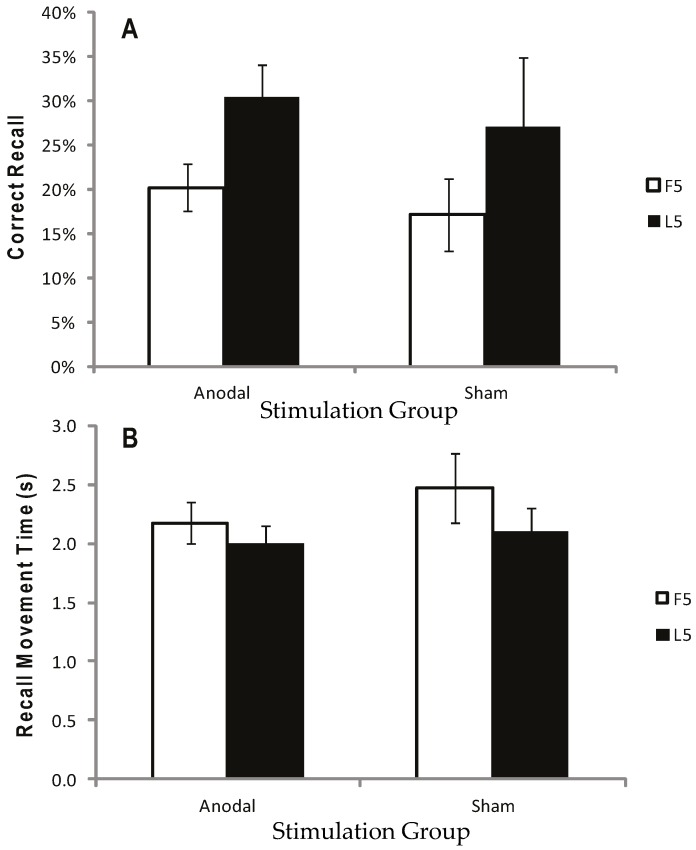
Correct recall and recall movement time showing no reliable difference between tDCS and sham for either the first five (F5) or last five (L5) trials. Measurements of Motor Learning in Experiment Two (First Five vs. Last Five Trials). Motor learning measures for the Anodal (atDCS) and Sham (S) stimulation groups averaged across F5 (white bars) and L5 (black bars) Test trials. (**A**) Proportion (%) of moves recalled in the correct sequential order (CR) (**B**) Mean time taken between moves during free recall (MT). Bars = standard error of the mean.

Participants remembered more of the sequence as the trials progressed and significantly more moves were recalled correctly in the F5 compared to the L5 trials (*F* (1, 15) = 15.79, *p* < 0.05, *η^2^_p_* = 0.51; Figure 6a). There was no main effect of stimulation group (*p* = 0.604) and no trial × stimulation group interaction (*p* = 0.953) (Figure 6a and Figure 7a).

Movement Recall Time improved steadily throughout the task (Figure 6b); hence there was a significant effect of trial on MT (*F* (1, 15) = 4.94, *p* < 0.05, *η^2^_p_* = 0.25), but no significant main effect of stimulation group (*p* = 0.450), and no trial × stimulation group interaction (*p* = 0.450) (Figure 6b and Figure 7b).

#### 2.2.6. Discussion

Older adults were able to learn a novel sequence of movements as the task progressed, and were capable of recalling just over one third of the 30-move sequence by the final Test trial. Participants also became quicker at recalling the moves by the time it got to the final five trials (i.e., main effect of trial on MT).

With regard to the effects of tDCS on learning, the results matched the findings of Experiment One—there was no clear benefit of tDCS. Anodal Stimulation (atDCS) did not increase the number of moves that older participants recalled correctly, or the speed at which they were able to recall them. The idea that tDCS might be more likely to improve learning in an older group (where learning ability was reduced) by increasing neuronal activity of M1 was therefore not supported.

## 3. General Discussion

Transcranial Direct Current Stimulation (tDCS) has previously been shown to modulate neuronal activity when applied over M1 [19,20,21,22,23,24,25,26,54]; and to improve performance of simple motor tasks [31,32,33,42,49] as well as some learning tasks [11,38,39,40,45,46,47,49,75,76], performed by healthy groups. While these results appear promising, the evidence-base for using tDCS as a rehabilitation therapy is still weak. Firstly, there are some null findings in the behavioural literature [58,59,69]. Secondly, only a few studies have examined the effects of tDCS on motor learning in groups of older adults, and these have often used coarse measures (e.g., global movement speed within the JTT [62]). Thirdly, there remains an ongoing debate about the neurophysiological effects of tDCS, with a recent meta-analysis suggesting that the only consistent effects are upon MEP amplitude [18] (though this review is controversial and there has been disagreement over interpretation of findings [77,78]).

In order to directly examine the impact of tDCS on real-world learning, we performed two experiments. Our first experiment tested young adults’ learning to make 32 aiming movements with their right hand whilst undergoing atDCS over the left M1, ctDCS over the right M1, or with Sham tDCS. Though participants clearly improved across the stimulation period (i.e., more of the moves were recalled correctly, and at a faster pace, as the task progressed) tDCS had no clear impact on performance or learning. This was true for both the atDCS and ctDCS groups, despite some of the past research suggesting otherwise [20,55,56]. Transcranial Direct Current Stimulation (tDCS) also had no effect on the way in which participants appeared to be encoding elements of the motor sequence—in the Transfer trial (i.e., where the location of targets was changed), all participants demonstrated a significant drop in speed and accuracy of recall compared to their scores in the final Test trial. This suggests that, regardless of the tDCS condition, participants learned the sequence spatially, rather than by using the characteristics of the targets themselves (e.g., colour and form). Independently, we have shown that when motor capacity is reduced, a person’s ability to memorise and reproduce a motor sequence is impaired [35]. We might, therefore, expect positive facilitating effects of tDCS on motor performance to manifest in improved ability to encode a novel motor sequence. However, no such effects were observed.

The failure of tDCS to enhance learning in Experiment One could have been caused by the age and skill level of our sample: there may have been little room to improve the functioning of the cortical networks involved in motor learning in healthy young adults. To address this point, we ran a second experiment using older adults, who would be expected to have poorer motor learning capacity and age-related deficits in motor performance, relative to the younger adults, and hence should be more likely to benefit from tDCS [11,35,65,66,67,73]. Experiment Two demonstrated that the older adults were indeed slower and less able to recall the sequences compared to the young, but all participants learned more of the sequence, and became quicker at recalling as the trials progressed. Critically, there were no significant effects of tDCS on learning. In this case, it cannot be argued that our task was not difficult enough or that the skill level of the participant group did not necessitate any ‘enhancement’, because even by the end of the task, older adults were only capable of recalling just over a third of the 30-item sequence.

It seems unlikely that the lack of tDCS effects in the present work could be attributed to our choice of parameters (such as current intensity, length of administration etc.). Most of the previously cited research showing positive tDCS effects administered stimulation over area M1 for 10–20 min with currents ranging between 1 to 1.5 mA. The parameters set in Experiments One and Two (i.e., 30 min at 1.5 mA) were not reduced compared to those applied by research groups that have found ‘enhancing’ tDCS effects in the past using these conditions [43,44,45,46,49,55,56,76]. Although these past studies involved stimulating M1, it could just be that stimulating this region may not have been optimal for improving learning in the tasks we used presently.

Both versions of our learning task (i.e., the Greek letters in Experiment One and the Arrows task in Experiment Two) have a strong working memory aspect, where elements of the sequence must be temporarily stored and manipulated during the formation of a new long-term representation [79,80,81,82]. Functional neuroimaging studies show that the dorsolateral prefrontal cortex (DLPFC) plays a predominant role in working memory processes [83], and that atDCS of the DLPFC can improve working memory performance on tasks that have some overlapping characteristics. For example, two studies presented letters on a screen and asked participants to press a button to indicate whether that letter had been shown three targets previously (the ‘three-back letter paradigm’) [84,85]. The study by Fregni et al. [84] showed that atDCS of the DLPFC improved performance on the three-back letter task, whereas atDCS of the M1 did not. In addition to mediating possible working memory contributions, tDCS focused on areas of the prefrontal cortex (PFC) may also aid sequence learning more generally. Studies using Positron Emission Tomography (PET) [86] and fMRI [87] have indicated PFC involvement in learning new sequences. It should be noted that Schendan et al. [87] also found evidence for Hippocampal/Medial Temporal involvement in sequence learning, in line with considerable existing evidence for a hippocampal contribution in memory for objects in spatial locations [88,89,90]. Thus, the spatial learning task presented here may well have an important hippocampal element.

It could be argued that a positive effect of tDCS on sequence learning may have been produced had the electrodes been positioned over the PFC (and perhaps specifically the DLPFC) rather than M1, but we believe that this would not be sufficient for benefiting the rehabilitation of motor sequence learning, because motor capacity is an essential part of being able to learn a new movement [35]. If a stroke patient has a motor deficit, they will be unable to physically carry out the movement required in order to learn a given action. Moreover, the literature strongly suggests that tDCS lacks focality and an electrode positioned over area M1 is likely to stimulate a network of brain areas with connectivity to this brain region [12,28,29,30]. This would imply that it should be possible to reach the other areas necessary for learning a new movement through stimulation of M1. Clearly, there is uncertainty regarding the specificity of tDCS— multiple studies have found the stimulating effects of tDCS to spread beyond directly beneath the electrode [24,28,29,30]. Nevertheless, some results would imply that the electrode has to be placed over a certain area of the brain to evoke an effect in a particular part of the body. One example is a study by Tanaka et al. [91] who found that stimulating the ‘leg area’ of the brain influenced leg movement but not movement in the hand. This may be due to the fact that tDCS provides an electrical field that passes through areas of the brain that lie between the anode and cathode, so that when the hand area of M1 is stimulated, the DLPFC and the leg area are also covered, but during stimulation of the leg area, tDCS does not have as much coverage over DLPFC and the hand areas.

Our own reasoning for choosing M1 as the target area for simulation in the present work was based on the successes of previous researchers, who have reported positive effects of tDCS on implicit motor learning [55] and the formation of motor memories [47]. The question of where to place tDCS electrodes in order to induce reliable effects is indeed an important one, and only adds to the confusion with regards to the optimal parameters required in order to evoke reliable effects using this technique.

Not only is the focality of tDCS uncertain, the claims made about the nature of the cortical effects induced by tDCS have been questioned in recent years. A systematic review of 30 experiments identified that tDCS was only capable of initiating consistent changes in one marker of cortical excitability: the Motor Evoked Potential (MEP) amplitude (the study by Nitsche and Paulus for example) [18]. This is usually measured using Transcranial Magnetic Stimulation (TMS) to generate an MEP after tDCS and then recording the changes in MEP amplitude via surface electrodes. Other markers included in the review did not replicate reliably across a 14 year period—for example, blood flow signals measured with Functional Magnetic Resonance Imaging (fMRI) did not consistently change post tDCS [57]. There are also behavioural studies that failed to identify any measurable effect of tDCS when completing complex motor learning tasks [58,59,69]. Unlike familiar actions, such as writing or tracing, learning a novel skill entails adopting new movement patterns in order to improve performance beyond one’s current capacity [92], and so could be argued to better match the processes occurring during rehabilitation.

For our research group, a primary driver for examining the effects of tDCS is to promote and enhance rehabilitation. If tDCS continues to produce inconsistent outcomes we must reconsider the efficacy of administering this technique in clinical settings. One should certainly question the therapeutic benefit of an intervention that is not always guaranteed to induce a positive outcome. Moreover, in cases where it does enhance, e.g., recovery of function, it only does so at a course level that is potentially driven by an ‘arousal effect’ (i.e., the stimulation increases concentration during mundane tasks). On the one hand, one could argue that any chance of improvement, even if only minor and general, could have the power to improve the autonomy and confidence of patients enough to have a positive impact on the health services in terms of assistance costs. A more important point, however, is that the disappointment yielded by ‘failed’ attempts at using tDCS in rehabilitative settings could damage patients’ recovery by deterring them from trying other options.

Moreover, in addition to being simply ineffective, there is also a concern that tDCS may actually do harm in some cases. The mild sensations frequently reported by those who have received tDCS have been described as ‘tingling’ or ‘itching’ feelings beneath the electrodes, and/or a mild headache. It has been implied that there is no evidence for significant negative side-effects when using tDCS with healthy individuals [93], but there are data that suggest tDCS can make motor symptoms worse in certain clinical groups; for example in cases of Writer’s Cramp and Musician’s Dystonia [94,95]. Furthermore, it has been shown that tDCS can yield unwanted changes in autonomic regulation—a problem that stroke patients are already at risk of developing [96,97]; including heightened sympathetic nerve activity and increased blood pressure flow to the muscles [95]. Heightened sympathetic nerve activity is in turn linked to disorders such as heart failure and obesity. The potential for tDCS to worsen symptoms, interact with pre-existing physiological problems (e.g., autonomic dysfunction following ischemic stroke) [97], and/or have no real benefit, is a cause for concern, especially because the evidence that tDCS can improve clinical outcomes remains unclear. In the case of stroke, some studies have simply failed to find any significant functional improvement [98,99]. Moreover, most studies which have found positive effects have involved very small groups of patients (*n* ≤ 15) in the chronic phase of stroke (typically left-hemisphere subcortical stroke) with mild-to-moderate motor impairment [22,33,100,101,102,103,104]. This research is often further limited by a tendency to rely on overly simplistic outcome measures (such as the speed of completing the subtests within the JTT) and a failure to compare the tDCS effect with the proven benefits of other approaches (e.g., CIMT). Note that the present research only aimed to recruit a small number of participants (albeit a similar number of subjects as cited in previous studies of this type [44,45,46,47,48,49,55]); which was based on our knowledge that there is questionable evidence with regards to the efficacy of tDCS (i.e., will it work?) and the safety of the method in clinical groups (i.e., could tDCS also cause harm in older adults who show age-related decline [65] in motor and cognitive abilities?). Our small group numbers are a limitation of the present work, but also a necessary ethical precaution. Most critically, the cases where tDCS has been found to evoke positive effects have involved groups of a similar, if not smaller, size [44,45,46,47,48,49,55].

## 4. Conclusions

Our work set out to clarify whether tDCS is capable of enhancing learning of a complex motor skill, particularly in older adults. We found no effect of tDCS on motor learning in healthy younger and older adults. One explanation for the inconsistent effects of tDCS is that the stimulation may produce a general ‘arousal effect’ that would only improve motor performance in tasks that otherwise are particularly likely to lead to boredom or fatigue. The tasks we used were complex and engaging and so there would have been little room for tDCS to increase participant engagement, and hence boost performance. An alternative argument could be that because the experiments in the present work demanded more than just M1 activity, and likely relied upon a complex network of neuropsychosocial processes, our set-up might not have ‘hit’ the best brain areas to enhance learning (despite the evidence to suggest that tDCS has widespread effects [19,24]). This is important to acknowledge when considering our argument with regard to the ineffectiveness of tDCS as a motor ‘facilitator’—the effects might indeed be task-specific [105]. Further clarification of whether tDCS can really improve movement control and/or learning (and via which optimal parameters [22]) without any long-term side effects is a priority. If reliable effects cannot be achieved with healthy participants in laboratory conditions, then it is unlikely that this tool would make a useful clinical contribution. Researchers should also use caution when applying tDCS to clinical groups—both for the sake of the patient (i.e., the possibility of worsening symptoms) and the health service (i.e., wasting limited resources on an ineffective therapy).
